# Supplementary material for: Osteopontin Splicing Isoforms Contribute to Endometriotic Proliferation, Migration, and Epithelial-Mesenchymal Transition in Endometrial Epithelial Cells
Source: Int J Mol Sci. 2022 Dec 5;23(23):15328. doi: 10.3390/ijms232315328 (PMC9738877; doi:10.3390/ijms232315328)
Supplement: Supplementary file 1 [file ijms-23-15328-s001.zip › ijms-2044282-supplementary.pdf]

Table S1. Patient clinical characteristics.

Patient No.	Age	Preintervention diagnosis	Lesion size	Classification/Staging (ASRM for endometriosis OR Sonographic Classification of Adenomyosis)	Ca-125 level
1	31	Myoma	95*43 mm		
2	33	Myoma	87*113*75 mm		
3	40	Myoma	12*12*13 mm		
4	41	Myoma	57*43*54 mm		
5	32	Myoma	57*82*54 mm		
6	38	Myoma	62*38*46 mm		
7	43	Myoma	36*73*120 mm		
8	35	Myoma	95*43*60 mm		
9	39	Myoma	87*75*103 mm		
10	32	Myoma	52*12*16 mm		
11	35	Myoma	95*48*60 mm		
12	41	Myoma	45*48*50 mm		
13	37	Adenomyosis	81*68*77 mm	Severe	102.4 U/mL
14	41	Adenomyosis	62*38*46 mm	Severe	36.2 U/mL
15	42	Adenomyosis	70*50*70 mm	Severe	36.08 U/mL
16	40	Adenomyosis	78*85*86 mm	Severe	20.3 U/mL
17	41	Adenomyosis	60*53*59 mm	Severe	31 U/mL
18	41	Adenomyosis	68*62*64 mm	Severe	67.83 U/mL
19	36	Adenomyosis	100*78*15 mm	Severe	140.1 U/mL
20	42	Adenomyosis	71*60*77 mm	Severe	
21	39	Adenomyosis	69*48*69 mm	Severe	75.02 U/mL
22	40	Adenomyosis	67*60*74 mm	Severe	
23	39	Adenomyosis	54*46*61 mm	Severe	64.72 U/mL
24	42	Adenomyosis	55*41*53 mm	Severe	
25	32	Ovarian Endometrioma	R 51*41*53 mm	III	23.39 U/mL
26	33	Ovarian Endometrioma	R 94*72*113 mm	IV	
27	32	Ovarian Endometrioma	Bilateral (R 76*57 mm, 42*45 mm, L 25*21 mm)	IV	109.7 U/mL
28	38	Ovarian Endometrioma	R 40*40 mm	III	99.96 U/mL
29	40	Ovarian Endometrioma	L 62*51*58 mm	III	19.92 U/mL
30	33	Ovarian Endometrioma	L 40*23*40 mm	III	
31	33	Ovarian Endometrioma	L 71*31*34 mm	IV	119.8 U/mL
32	33	Ovarian Endometrioma	R 71*34*32 mm	III	119.9 U/mL
33	35	Ovarian Endometrioma	Bilateral (58*37*67 mm and 46*36*61 mm)	III	23.39 U/mL
34	32	Ovarian Endometrioma	L 56*36*53 mm, 47*42*50 mm	III	99.96 U/mL
35	28	Ovarian Endometrioma	Bilateral (R 52*44 mm, 10*9 mm, 15*14 mm, L 53*39 mm)	IV	109.7 U/mL
36	41	Ovarian Endometrioma	L 40*23*40 mm	IV	19 U/mL

Table S2. The oligonucleotide sequences of primer pairs used in this study.

ID	Accession Number	Primers	Oligonucleotide Sequence 5'- 3'	PCR products Base Pair
qOPN	A. NM_001040058.1	Forward	CACCTGTGCCATACCAGTTAA	531 bp
	B. NM_000582.2	Reverse	GGTGATGTCCTCGTCTGTAGCATC	489 bp
	C. NM_001040060.1			450 bp
OPNa	NM_001040058.1	Forward	GGATCCCTCACTACCATGAG	903 bp
		Reverse	AAGCTTGACCTCAGAAGATGCACT	
αV integrin	NM_002210	Forward	GACTGTGTGGAAGACAATGTCTGTAAACCC	304 bp
		Reverse	CCAGCTAAGAGTTGAGTTCCAGCC	
β3 integrin	NM_000212	Forward	GTGCTGACGCTAACTGACC	284 bp
		Reverse	CATGGTAGTGGAGGCAGAGT	
CD44 standard	NM_000610	Forward	CAACTCCATCTGTGCAGCAAA	300 bp
		Reverse	GTAACCTCCTGAAGTGCTGCTC	
CD44 variant	NM_000610	Forward	AACGCTTCAGCCTACTGCAAA	127 bp
		Reverse	TCTTCCAAGCCTTCATGTGATG	
E-Cadherin	NM_004360	Forward	TCCCATCAGCTGCCCAGAAA	500 bp
		Reverse	ATGACTCCTGTGTTCCTGTTA	
N-Cadherin	NM_001792	Forward	CACTGCTCAGGACCCAGAT	415 bp
		Reverse	TAAGCCGAGTGATGGTCC	
VCAM1	NM_001199834	Forward	CCGGAAGGTGTATGAACTG	431 bp
		Reverse	TCCATGGTGATCTCTCCTC	
ICAM1	NM_000201	Forward	GTCCCCCTCAAAAGTCATCC	942 bp
		Reverse	AACCCCATTCAGCGTCACCT	
β actin	NM_001101.3	Forward	CGG ATG TCC ACG TCA CAC TT	500 bp
		Reverse	GTT GCT ATC CAG GCT GTG CT	

Table S3. Single stranded RNA oligonucleotides of OPN and CD44v used for knockdown study.

siRNA		Oligonucleotide Sequence 5′-3′
siOPN	Sense	GGUCAAAAUCUAAGAAAGUUTT
	Antisense	GCUGACCUCUGCAAGGCUUUCAAUA
siCD44	Sense	CAUCGGAUUUGAGACCUGCAGGUAU
	Antisense	AUACCUGCAGGUCUCAAAUCCGAUG
